# Characteristics, Motivation, and Challenges of Global Research on Dengue Vaccination

**DOI:** 10.1002/rmv.70122

**Published:** 2026-03-01

**Authors:** Doris Klingelhöfer, Markus Braun, Isabelle M. Kramer, Christina A. Naser, David A. Groneberg, Dörthe Brüggmann

**Affiliations:** ^1^ Institute of Occupational Social and Environmental Medicine Goethe University Frankfurt Frankfurt Germany; ^2^ The Unit of Entomology Department of Biomedical Sciences Institute of Tropical Medicine Antwerp Belgium; ^3^ Paul Ehrlich Institute Langen Germany

**Keywords:** aedes aegypti, mosquito‐borne disease, prevention, research output, zika virus, zoonosis

## Abstract

Due to the sharp increase in dengue infections in recent years, developing effective and regionally adapted vaccines is imperative. This requires research strategies aimed at vaccines that are free of side effects and effective for all four serotypes. Currently, two vaccines are licenced in some countries, each with certain restrictions. Advanced vaccines are in demand. This study, therefore, analyzes what has been done so far, by whom, and under what conditions. Who has benefited, and where is there an urgent need for further research? An advanced strategy with chronological, geographical, and socio‐economic patterns was applied to answer these questions. A total of 1758 publications and 137 clinical studies were identified. The results show that the pharmaceutical industry dominates the research both in their countries of origin and in their study sites. Sustainable collaboration between academic institutions, industry, and government agencies is crucial for research on DENV_VAC._ However, this generally applies to vaccine research, in which universities and industry play an important role. Depending on historical networking, better or worse conditions have developed for regional research. The Global South is exemplified by South American nations like Brazil, which is actively developing its vaccine, as well as by Southeast Asian countries such as India, which is also pursuing the development of its vaccine. Africa is not represented in the research, although there is evidence of virus circulation and a potentially underestimated disease burden. Financial support comes mainly from government agencies, except in France and Japan, where industry sets the research framework. This results in a clear regional concentration of research, which can be explained by the lack of access to reliable monitoring and serological data, but also by the regional interests of pharmaceutical companies, their resources, and financial support. This is what future research must be concerned with.

**Abbreviations**
ADE: Antibody‐dependent Enhancement
Ae: Aedes
AFRIMS: Armed Forces Research Institute of Medical Science, Thailand
CDC US: Centre for Disease Control and Prevention
CIBG: Centre for Genetic Engineering and Biotechnology, Cuba
DEMP: Density‐Equalising Map Projection
DENV: Dengue Virus
DENV_VAC_
: Dengue Vaccination
DHF: Dengue Haemorrhagic Fever
DSS: Dengue Shock Syndrome
EC: European Commission
ECDC: European Centre for Disease Prevention and Control
FDA: US Food and Drug Administration
GDP: Gross Domestic Product
HI: High‐Income country
IF: Impact Factor
IgG: Immunoglobulin G
IVI: International Vaccine Institute
JAMA: Journal of the American Medical Association
LATV: Live‐attenuated Tetravalent Vaccine
LMI: Lower‐Middle‐Income country
NEJM: New England Journal of Medicine
NewQIS: New Quality and Quantity Indices in Science
NIAID: National Institute of Allergy and Infectious Diseases, USA
NIH: National Institutes of Health, USA
UIS: UNESCO Institute for Statistics
UMI: Upper‐Middle‐Income country
UNESCO: United Nations Educational, Scientific and Cultural Organization
WHO: World Health Organization
WoS: Web of Science

## 1 Introduction

The dengue infection figures for the first half of 2024 already exceed those of the record year 2023, with over 10 million cases already reported from 176 countries. Most cases occur in the Americas. [1] The rising number of dengue infections and the associated burden led the *World Health Organization* (WHO) in 2021 to classify it as one of the greatest threats to global health. [2] Dengue fever is the most common and fastest‐spreading mosquito‐borne disease in tropical and subtropical regions. During the last 20 years, the number of reported cases has at least decupled, as has their geographical spread.

The global incidence has increased more than thirtyfold in the last 50 years in many countries, especially among children. [3, 4] About half of the world population is at risk, with the number of annual infections estimated at between 100 and 400 million. [5].

The growth is mainly man‐made, resulting from climate change, urbanisation, and intensified international trade and travel. [1] As climate change makes conditions increasingly favourable for the main vector, the mosquito *Aedes (Ae.) aegypti*, the outbreak region at risk is becoming larger. [6] Once infected, a mosquito can transmit the virus for the rest of its life. [7] According to the WHO, dengue fever is currently spreading to temperate zones. Europe is, therefore, also susceptible to an outbreak. [5].

Dengue fever is endemic in more than 100 countries. So far, most cases of infection have been reported in 2023. [7] In the first half of 2024, 7000 deaths were already reported from 84 countries or regional territories, twice as many as in the same period in 2023. Most cases have been reported from Brazil, followed by other South American countries. Infections have also been reported in Europe, including autochthonous cases. In 2024 alone, 129 autochthonous cases were reported in Italy, 76 in France, and 8 in Spain (data until October 8, 2024). [8, 9].

As many countries do not have adequate monitoring systems, the burden of DENV is underestimated worldwide. [10] Therefore, the actual figures are certainly much higher than those reported, also due to misdiagnoses and the large number of asymptomatic cases. [11] Infections place an extreme burden on healthcare systems due to the costs incurred, prolonged hospitalisation, and loss of productivity. [12].

The DENV infection is often asymptomatic or clinically inapparent. [11, 13] However, DENV infection can also lead to severe illness or even death, mostly during second infections. As a result, more severe fevers occur in regions where DENV is circulating. [14] High fever, pain, nausea, swollen glands, and rash are the most common mild symptoms. The more severe and sometimes fatal manifestations, like severe dengue haemorrhagic fever (DHF), life‐threatening dengue shock syndrome (DSS), and dengue encephalitis, often occur when the first symptoms are gone. [7].

The growing pressure of disease burden and spreading make preventive measures urgently necessary. The best‐known recommendations for avoiding mosquito bites are to use insect repellent, wear loose clothing, avoid endemic regions when travelling, seek out ventilated places, and control the spread of mosquitoes privately. [15] In addition to chemical control by spraying with insecticides, which has proven to be insufficient for *Ae*. *Aegypti* and *Ae. Albopictus* due to their resistance, for example, to pyrethroids, and whose propagation path is still unknown, biocontrol approaches were presented. [16] A reduction of the DENV virus can also be achieved by novel methods that limit the ability of mosquitoes to transmit the DENV virus, for example, by releasing mosquitoes infected with the endosymbiont *Wolbachia pipientis* or by using genetically modified mosquitoes, which could also lead to a reduction in the mosquito population. [17] Facilitating the introduction of the endosymbiont through the drone‐assisted release of mosquitoes appears promising. [16] Reduction of breeding sites in conjunction with the information and mobilisation of the population and stakeholder groups is also recommended. However, many methods show despite a reduction in the vector density, no considerable impact on the disease burden so far. [18].

Due to the shortcomings of these prevention strategies, the development and use of vaccines are crucial. Severe forms of disease occur in children with both primary and secondary dengue infections. Vaccine development, especially for children with incompletely formed immune systems, is essential, but also an extraordinary challenge. [19].

Two dengue vaccines are currently authorised worldwide. *Sanofi Pasteur* (France) developed the first, which was authorised in 2015 under the name *Dengvaxia* (CYD‐TDV). It is based on a yellow fever backbone, while the latter, in 2023, licenced a two‐dose vaccine *Qdenga* (*Takeda*, Japan ‐ TAK‐003), which was developed on a DENV‐2 backbone basis with recombinant strains that express the surface proteins for DENV‐1, ‐3, ‐4. [20].

The *National Institutes of Allergy and Infectious Diseases* (NIAID) of the National Institutes of Health (USA) has a third vaccine (TV‐005) that has nearly completed Phase 3 clinical trials. [21] All three vaccines are mono‐component, live‐attenuated tetravalent vaccines (LATV).

Currently, the market leader is TAK‐003 (Qdenga). It does not require pre‐infection and is recommended for children aged 6–16 years in areas of high transmission. [22] Dengvaxia, the only vaccine licenced in the USA, [23] will be discontinued in 2026. [23].

However, there are some contraindications for the vaccination. [22] All previously developed vaccines had shortcomings, which makes it clear that there was incomplete knowledge about dengue immunity when the vaccines were developed. [3] Dengue vaccine development was and is still very difficult, as it must protect against all four serotypes.

In addition, *antibody‐dependent enhancement* (ADE) in secondary infections must be prevented. [24] The antibodies produced by the tetravalent vaccination are usually cross‐reactive and their protective phase lasts only a short time, so subsequent infections with a different serotype can exacerbate the disease. ADE is the main reason for this. It is mainly induced by immunoglobulin G (IgG). Neutralising antibody titres alone are, therefore, not sufficient to protect against symptomatic infections. The mechanisms of ADE are still not fully understood, although the link with secondary severe dengue infection was established some 60 years ago. [25] Previous studies question this strong link and urge the development of a vaccine for a dengue‐naïve population. [19] Nevertheless, a vaccine‐induced antibody must be free of ADE to avoid escalation of the disease. [25].

Meta‐analyses have shown the efficacy and immune response of the authorised vaccines. However, safety concerns require further evaluation. [26] The results of previous studies underline the need for personalised vaccination strategies tailored to an individual's serostatus to optimise the efficacy and safety of vaccination. [27] The development of a new tetravalent nanoparticle vaccine has proven successful in mouse experiments, as it induces immunity without ADE. [28] Also, the development of subunit vaccines with priming‐boosting strategies could help to prevent ADE by following the responses to natural infections. Antibody engineering for the improvement of efficacy by reducing ADE or increasing the half‐life and effector functions could also improve outcomes. In addition, the use of antibody cocktails may also reduce the neutralising escape variant and thus be effective against the different serotypes without ADE. [25].

In addition to chimaeric attenuated live vaccines, other methods, such as mRNA/DNA‐based and recombinant subunit proteins, have also been used as antigens. The peptides enable the initiation of certain immune responses and thus reduce the risk of autoimmune reactions. However, as their own immunogenicity is weak, they must be accompanied by an adjuvant that has a stimulating effect. Neither of the two approaches led to the authorisation of a vaccine. [29].

The noticeable impact of climate change on the spread of dengue vectors meets fragile healthcare systems and political instability. Population and vector movements are among the consequences that favour the spread to other countries. Lack of or inadequate surveillance leads to false or delayed reports and their misinterpretation. [10] As this leads to an enormous burden for societies worldwide, the availability of regionally effective, safe vaccines authorised for all age groups is imperative. To meet this challenge, regional and target group‐oriented research is needed that does justice to everyone. The aim of this study is therefore to provide a detailed overview of the global dengue vaccine (DENV_VAC_) research and publication landscape. It will provide useful information to all stakeholders for the successful development of future comprehensive research strategies to combat dengue fever outbreaks worldwide that are ethical, equitable, and affordable.

## 2 Methods

### 2.1 Methodological Platform and Data Source

This study utilises the established analysis tools of the *New Quality and Quantity Indices in Science* (NewQIS) platform. [30] The standard techniques are further developed here and adapted to the topic of *dengue vaccination* (DENV_VAC_). The inclusion of established bibliometric methods and visualisation with *Density‐Equalising Map Projections* (DEMPs) [31] was applied for the first time in NewQIS. By default, all NewQIS studies use the *Web of Science* (WoS) Core Collection as a data source. It is one of the most comprehensive online databases for scientific literature and provides citation numbers for all listed issues. WoS‐indexed journals undergo a selection process. They must complete the peer review process, have an annually updated *Impact Factor* (IF), or be listed as an “Emerging Journal” to be indexed by WoS.

### 2.2 Search Strategy and Data Processing

Here, the search strategy had to be an elaborate string. It aims to maximise the number of publications related to the topic and minimise the number of publications not related. The best results were obtained with the following strategy combining synonyms of dengue with those of vaccination. The reduction of false entries was achieved by the title search for the dengue terms and the search in the title or author keywords for the vaccination terms.

Title: “Dengue*” OR “Break Bone Fever*” OR “Breakbone Fever*” OR “DENV‐1” OR “DENV‐2” OR “DENV‐3” OR “DENV‐4” OR “DENV1” OR “DENV2” OR “DENV3” OR “DENV4”

And

Title OR Author Keywords: (“vaccin*” OR “prevaccin*” OR “immuniz*” OR “immunis*” OR “inoculat*” OR “Dengvaxia” OR “Qdenga” OR “CYD‐TDV” OR “TAK‐003” OR “TV003” OR “TV005“ OR “TV003/TV005“ OR “DENVax “ OR “PIV “ OR “V180” OR “DPIV” OR “DSV4” OR “D1ME100” OR “TVDV” OR “LATV” OR “TDEN” OR “E80‐mRNA” OR “jab”)

The metadata of the publications was retrieved from WoS on 07/12/2024. They were stored in an MS Access database. The data on the institutions and sponsors had to be standardized manually, as the affiliation specifications and the designation for funders or grants are very different in the authors' entries. For further processing, the data were pooled and sorted according to the parameters to be analysed.

The articles that refer to clinical trials were searched for in *PubMed* using the following search term: (Dengue[Title] OR (DENV1[Title] OR DENV2[Title] OR DENV3[Title] OR DENV4[Title] OR DENV‐1[Title] OR DENV‐2[Title] OR DENV‐3[Title] OR DENV‐4[Title]) AND (Vaccine*[Title]). The entries found were filtered according to the article‐type Clinical study.

### 2.3 Data Analysis

This study integrates a variety of analyses ranging from chronological and geographical to epidemiological parameters. The temporal development of the publication and citation figures and the contribution of the countries with the most publications are at the centre of attention, as is the extended analysis of the countries of origin according to their socio‐economic and disease‐related characteristics. The DEMP method, which is based on an algorithm that distorts the country sizes according to the parameter value to be analysed, was used in part to visualise the country parameters. [31] This method is based on the physical principle of osmotic density equalisation, which results in countries with high parameter values being enlarged in size and countries with low values being reduced.

The countries of origin had to be updated and assigned to a current list of countries and regional areas for geographical demarcation. The assignment of the author affiliation to a superordinate unit or a country of origin must be done manually, as the affiliations are very different, and some institutions operate worldwide, particularly in the case of pharmaceutical companies. Therefore, the regional locations were considered in the country analyses, while the organizations' headquarters were used to assess the financing and networking of the institutions. Collaboration exists at the national level if at least two countries are involved in a publication, and at the institutional level, at least two different affiliations.

The funding data was collected from the WoS entries of the funding organizations and manually corrected and standardized at great expense to make it usable for analysis.

The data on clinical trials were taken from *ClinicalTrials.gov* and served as the basis for the interpretation of the publications based on it. Data on study type, year of study start, locations, eligible age of subjects, and vaccine type were used to describe the studies and compare them with the resulting publications.

### 2.4 Limitations and Strengths

The methodology used has some limitations that need to be mentioned. Although an established and advanced approach is applied that provides a reliable and valid data set for the evaluation of publications on DENV_VAC_, there are limitations in terms of the completeness of the metadata included. The entries of the online database WoS, one of the most prominent scientific literature databases, are limited to its indexed journals. Therefore, not all published articles can be found per se. WoS is also dominated by articles in English. However, the advantages of using WoS outweigh the disadvantages. It not only provides citation figures for each entry but also sets disciplinary requirements for the indexed journals, which guarantee a certain level of quality. In addition, it is generally not possible to find all entries using the search strategy in WoS. It is always a balancing act between maximising related entries and minimising unrelated entries. As a consequence, this approach limits the total number of articles included in the analysis database.

Citation parameters can only indicate how much attention an article or a national performance on a particular scientific topic has received in the scientific community. They are not a one‐to‐one measure of the quality of research. Furthermore, the values are susceptible to errors and misconduct, such as self‐citation. This must be considered when discussing the results of the citation analyses.

## 3 Results

The metadata of 1870 publications (*n*) on DENV_VAC_ could be retrieved by applying the search strategy from WoS. The majority (*n* = 1006, 53.80%) were published as original articles, 209 articles as reviews, and 114 documents as editorial material. A smaller number were published as news items, letters, progress reports, etc. Almost all publications found were written in English (*n* = 1850, 98.93%) and only a few in Spanish, French, German, and Norwegian.

### 3.1 Development Over Time

**FIGURE 1:**
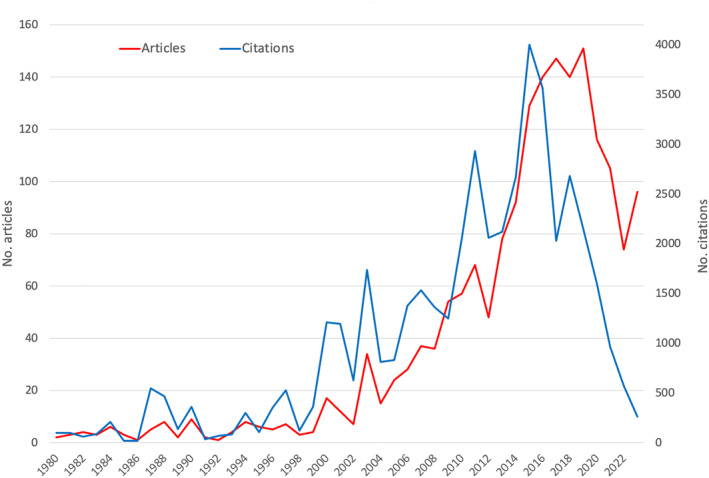
Development of the annual publication and citation numbers on DENV_VAC_ from 1980 to 2023.

The first publication about DENV_VAC_ indexed in WoS was published as editorial material in 1945 in the *Journal of the American Medical Association* (JAMA). From this point onwards, there was a steady stream of one or a low single‐digit number of publications per year. However, almost all of these were recognized, as evidenced by the high number of citations per article. It was not until 2000 that the annual publication figures reached double figures. It grew steadily in the following years, until 2019, the year with the most publications, only to fall again afterwards. Some annual peaks were observed: 2000 (*n* = 17), 2003 (*n* = 34), 2011 (*n* = 68), 2017 (*n* = 147), 2019 (*n* = 151). The annual citation figures developed similarly to the publication figures, also with some notable peaks: 187 (*c* = 546), 2000 (*c* = 1208), 2011 (*c* = 2932), 2015 ‐ the year with the highest number of citations to date (*c* = 3999). The subsequent downward trend was interrupted by an interim high, but at a lower level, in 2018 (*c* = 2681) (Figure 1).

Table 1 lists the most frequently cited publications on DENV_VAC_.

**TABLE 1:** The most‐cited publications on DENV_VAC_. NEJM = New England Journal of Medicine, Lancet = The Lancet, IF = WoS Impact Factor (2023).

Authors (country of origin of first author)	Year	Citations	Title	Journal	If
Hadinegoro et al. (Indonesia) [32]	2015	763	Efficacy and long‐term safety of a dengue vaccine in regions of endemic disease	NEJM	96.2
Capeding et al. (Philippines) [33]	2014	729	Clinical efficacy and safety of a novel tetravalent dengue vaccine in healthy children in Asia: a Phase 3, randomised, observer‐masked, placebo‐controlled trial	Lancet	98.4
Villar et al. (Colombia) [34]	2015	681	Efficacy of a tetravalent dengue vaccine in children in Latin America	NEJM	96.2
Sabchareon et al. (Singapore) [35]	2012	634	Protective efficacy of the recombinant, live‐attenuated, CYD tetravalent dengue vaccine in Thai schoolchildren: a randomised, controlled phase 2b trial	Lancet	98.4
Sridhar et al. (France) [36]	2018	478	Effect of dengue serostatus on dengue vaccine safety and efficacy	NEJM	96.2
Whitehead et al. (USA) [37]	2007	450	Prospects for a dengue virus vaccine	Nature reviews microbiology	62.2
Beatty et al. (USA) [38]	2015	338	Dengue virus NS1 triggers endothelial permeability and vascular leak that is prevented by NS1 vaccination	Science translational medicine	15.8
Murphy, whitehead (USA) [39]	2011	337	Immune response to dengue virus and prospects for a vaccine	Annual review of immunology	26.9
Johnson, roehrig (USA) [40]	1999	289	New mouse model for dengue virus vaccine testing	Journal of virology	4.0
Kinney et al. (Thailand) [41]	1997	268	Construction of infectious cDNA clones for dengue 2 virus: Strain 16,681 and its attenuated vaccine derivative, strain PDK‐53	Virology	2.8

#### 3.1.1 Countries' Contribution

**FIGURE 2:**
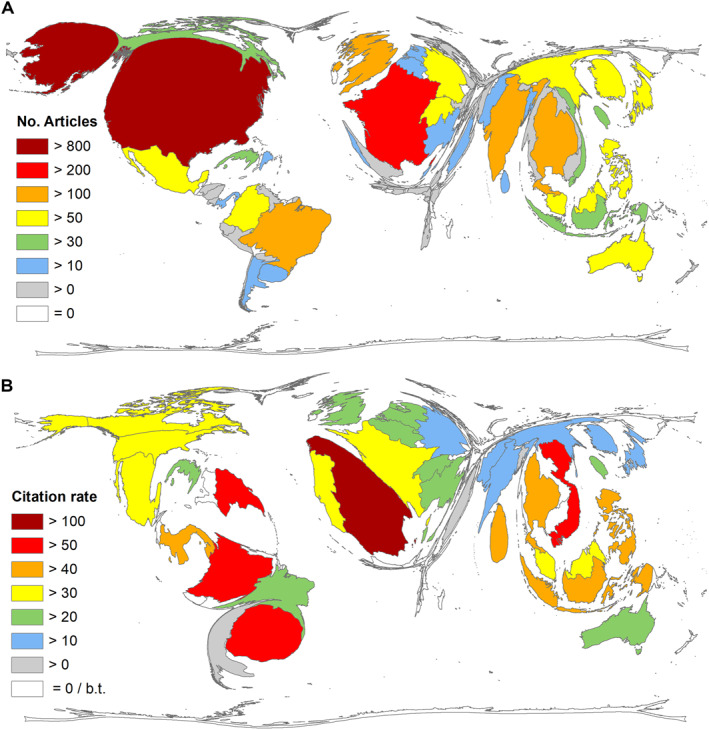
Countries' publication performance on DENV_VAC_. A) Publication numbers per country. B) Citation rate (threshold: 10 publications per country), b.t. = below threshold.

A total of *n* = 1758 publications (94%) could be assigned to a country of origin and thus included in the country analyses, with 83 countries publishing on DENV_VAC_. The country with the most publications was the USA, with *n* = 873 articles. France followed with less than a third (*n* = 235), followed by Thailand (*n* = 182), Singapore (*n* = 155), and Brazil (*n* = 145), to name the top 5 publishing countries (Figure 2A). The same order emerges if one looks at the national citation figures that the publications on DENV_VAC_ received: USA (*c* = 29,612), France (*c* = 8664), Thailand (*c* = 7795), Singapore (*c* = 6116), Brazil (*c* = 3448). A different ranking results if the publishing countries are sorted according to their citation rate, with an analysis threshold of at least 10 articles on DENV_VAC_. Here, Spain leads with a citation rate (cr) of 109.20, followed by Uruguay (cr = 87.04), Vietnam (cr = 56.12), Colombia (cr = 54.91), and the Dominican Republic (cr = 54.08) (Figure 2B).

**FIGURE 3:**
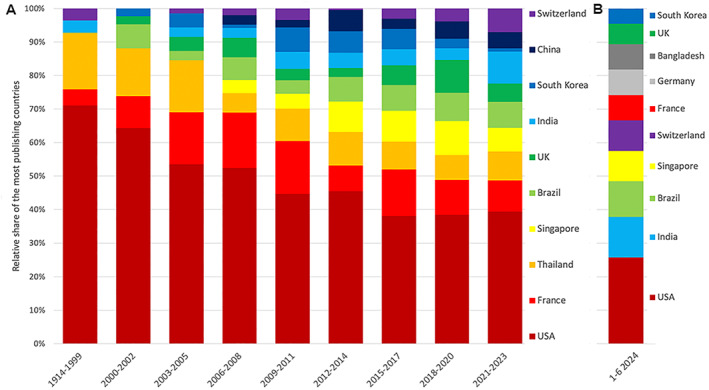
Development of the relative share of the overall most publishing countries from 1914 to 2023 in 3‐year intervals, except the first interval, which covers 1914 to 1999, and the most publishing countries from January to June 2024.

The contribution of the countries with the most publications varies over time (Figure 3A), with the USA's share decreasing while India's and Switzerland's shares increase. Looking at the first half of 2024, the order of countries with the most publications has changed: India, Brazil, Singapore, and Switzerland currently follow the USA (Figure 3B).

**FIGURE 4:**
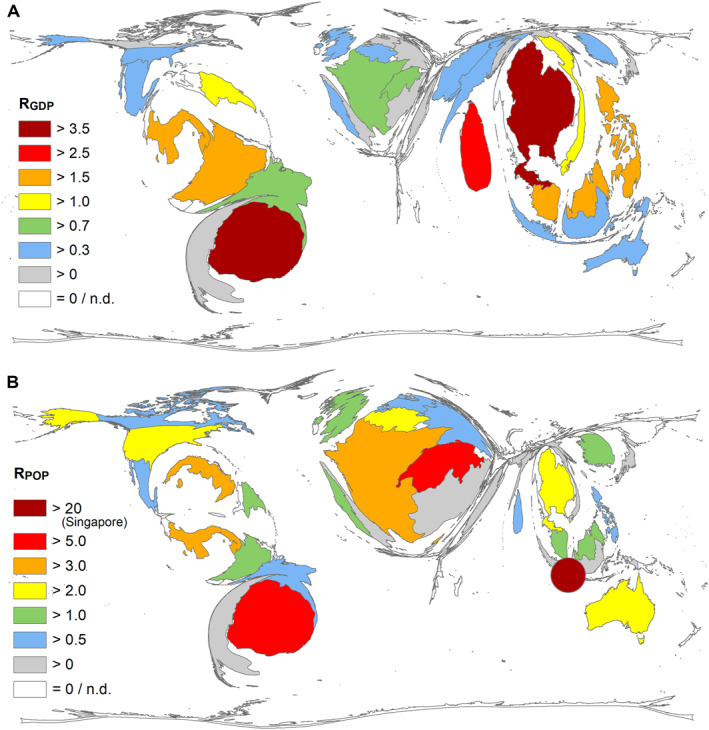
Countries' socio‐economic ratios (threshold: 10 publications on DENV_VAC_ per country). (A) *R*_GDP_ = number of articles/gross domestic product (GDP) in 10 billion US dollars. (B) *R*_POP_ = number of articles/population in a million. For Taiwan, no data is available. Singapore is shown as a point due to the limited possibilities of the mapping application, n.d. = not determined.

### 3.2 Socio‐Economic Indicators

When calculating the quotients of the number of publications and the countries' GDP (R_GDP_) and population size (R_POP_), other orders emerge (Supplementary Tab. 1).

1. R_GDP_ (no. Articles/GDP in billion US dollars): Uruguay, Thailand, Singapore, Sri Lanka, Panama (Figure 4A)
2. R_POP_ (no. Articles/Population in a million inhabitants): Singapore, South Korea, Portugal, Canada, Germany (Figure 4B).

#### 3.2.1 Collaboration at the International Level

**FIGURE 5:**
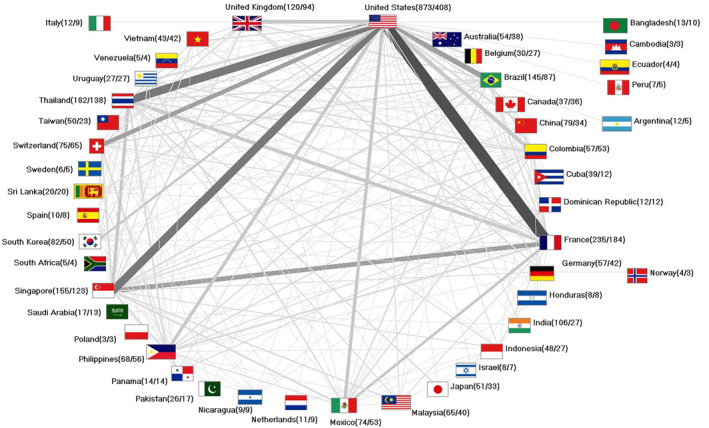
International collaborations. Numbers in brackets (number of publications/number of collaboration articles).

The analysis of publications with at least two affiliations from different countries revealed that *n* = 667 publications on DENV_VAC_ were produced as collaborative articles (37.94% of all articles assigned to a country of origin), predominantly as binational partnerships (*n* = 391). The most fruitful cooperation was between the USA and France (*n* = 121), followed by the USA and Thailand (*n* = 87), the USA and Singapore (*n* = 85), and the USA and Brazil (*n* = 55), meaning that the USA is at the centre of international partnerships (Figure 5). However, its share of cooperation at the international level is comparatively low (46.74%). The strongest partner countries, in contrast, have higher percentages to this effect: France (78.30%), Thailand (75.82%), Singapore (79.35%), and Brazil (60%).

### 3.3 Research Affiliations

Pharmaceutical companies and institutions involved in the development of DENV vaccines include the most publishing companies: (1) Sanofi Pasteur (France, n = 202), (2) National Institutes of Health (NIH), mostly National Institute of Allergy and Infectious Diseases (NIAID) (USA, n = 125), (3) Takeda Pharmaceutical (Japan, n = 77), and 4) GlaxoSmithKline Inc. (UK, n = 32). In addition, there are the research institutes of the armed forces of the USA (Walter Reed Army Institute of Research, n = 124; US‐Army n = 35) and Thailand (Armed Forces Research Institute of Medical Science – AFRIMS, n = 42). Otherwise, universities from various countries are represented in the list of affiliations with at least 30 articles: USA (Johns Hopkins University, n = 117, University of North Carolina, n = 58), Thailand (Mahidol University, n = 103), UK (University of London, n = 54), Brazil (University of Sao Paulo, n = 48), and Singapore (Duke‐NUS Medical School, n = 44, National University Singapore ‐ NUS, n = 43). Cuba is represented by the Centre for Genetic Engineering and Biotechnology (CIBG) with n = 32 publications and South Korea with the headquarters of the International Vaccine Institute (IVI) (n = 42). The US Centre for Disease Control and Prevention (CDC) contributed n = 58 publications.

**FIGURE 6:**
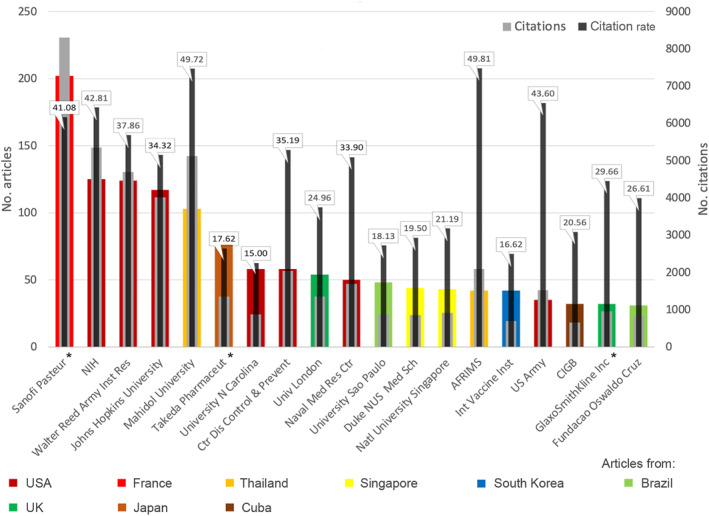
Most publishing institutions (threshold: 30 publications on DENV_VAC_, * For companies with branches in several countries, the total is calculated, and the country of origin is indicated as the country of the headquarters. AFRIMS = Armed Forces Research Institute of Medical Science, CIGB = Centre for Genetic Engineering and Biotechnology, NIH = National Institutes of Health, Inst Res = Institute of Research, N Carolina = North Carolina, Ctr Dis Control & Prevent = Centre for Disease Control & Prevention, Univ = University, Naval Med Res Ctr = Naval Medical Research Centre, Duke NUS Med Sch = Duke‐NUS Medical School, Natl = National, Int Vaccine Inst = International Vaccine Institute.

The number of citations received by the publications of highly committed institutions varies greatly. When calculating the citation rate, it becomes clear that the Thai institutions are in the lead with citation rates of almost 50. The US Army and the NIH also achieved high citation rates of over 40, while *Takeda Pharmaceutical* in Japan only achieved a citation rate of 15 (Figure 6).

The strongest collaboration at the institute level took place between *Johns Hopkins University* and the NIH (*n* = 69), which also cooperates noticeably with the universities of Vermont and North Carolina (threshold value 10). Another US‐American network exists between the *Walter Reed Army Institute of Research* (WRAIR) and various institutions of the US Army, the research institute of the Thai armed forces, and *GlaxoSmithKline* (UK). *Sanofi Pasteur* collaborated with Philippine, Mexican, and Thai research institutes or universities, as well as with the *University of Washington* and the affiliated *Fred Hutchinson Cancer Centre*. Another international partnership at the university level was between Germany and the UK.

### 3.4 Research Funding

National governments, universities, private companies, foundations or charities, and other organizations and institutions participated in funding research on DENV_VAC_. A total of 42 countries provided grants (g), with 39 governments and universities or research institutes from 27 countries participating. The USA provided the most funding (*g* = 621), followed by Brazil (*g* = 143), France (*g* = 130), China (*g* = 111), and Japan (*g* = 110) (Table 2).

**TABLE 2:** National funding from countries with at least 10 DENV_VAC_ grants. Govt = Government, Co. = Company, Univ. = University, Res. Inst. = Non‐university research institutes, Fdn = Foundations, NPO = Non‐profit organizations, Soc. = Societies, Assoc. = Associations, TU = Trade unions, * = National academies and universities are assigned to government funding.

Country	Total	Govt	Pharma Biotech IT Co.	Univ.*, Res. Inst.	Trusts, Fdn, NPO	Soc., Assoc., TU
USA	621	466	38	43	73	1
Brazil	143	140	0	2	1	0
France	130	10	111	3	6	0
China	111	109	1	1	0	0
Japan	110	58	43	6	2	1
UK	70	50	4	6	4	6
India	61	52	5	4	0	0
Thailand	55	33	0	21	1	0
South Korea	53	43	0	10	0	0
Singapore	39	34	0	4	1	0
Taiwan	35	31	0	4	0	0
Malaysia	29	6	0	23	0	0
Mexico	23	15	0	7	1	0
Cuba	22	14	4	0	4	0
Portugal	20	15	0	5	0	0
Germany	19	9	5	3	2	0
Canada	18	13	0	3	2	0
Indonesia	17	13	0	4	0	0
Australia	15	10	0	4	1	0

The proportion of government funding varied. The largest share of government grants came from Brazil and China. The US government provided around three‐quarters of all grants, while Japan only financed around 50%. In France, the government was only marginally involved in DENV_VAC_ research funding. The other French and Japanese grants came from the pharmaceutical companies developing the vaccines: Sanofi Pasteur and Takeda. In the USA, the governmental NIH, in particular the NIAID, is involved in the development of a vaccine that is currently in the final phase of clinical trials. International organizations (*g* = 42) also funded research on DENV_VAC_. The *European Commission* (EC) provided the largest share (*g* = 26). The WHO contributed 9 grants.

The highest proportion of grants per article (G/A) (threshold 10 articles) was in Japan (G/A = 2.16), followed by Portugal (G/A = 1.67), China (G/A = 1.41), Brazil (G/A = 0. 99), and the USA (G/A = 0.71). The lowest values were recorded by Switzerland (G/A = 0.08), the Philippines (G/A = 0.09), the Netherlands (G/A = 0.09), Belgium (G/A = 0.10), and Colombia (G/A = 0.12).

### 3.5 Publication Patterns Based on Clinical Studies

The publications refer to 137 clinical trials (ct) registered at clinicaltrials.gov. Of these, ct = 106 are completed, and ct = 48 have results. There are currently 17 trials that are not yet recruiting or recruiting (until evaluation date: August 2024). Nine studies have been withdrawn or cancelled, or their status is unknown. Different study phases were processed in the completed studies: Phase 1 (ct = 52), Phase 2 (ct = 36), and Phase 4 (ct = 15). Most were interventional studies (ct = 100), only a few were observational studies (ct = 6), and only one was a patient registry. The sponsors were the developing companies (ct = 67), US federal institutions (ct = 50), and others, such as universities and organizations (ct = 26).

Based on the search strategy, 142 publications on these clinical trials (n_CT_) were found. The authors come from 32 countries, with the USA having the most publications. Looking at the affiliation without the authors from industry, Thailand (*n*_CT_ = 36), the Philippines (*n*_CT_ = 17), Colombia (*n*_CT_ = 17), and Brazil (*n*_CT_ = 15) are at the top. Other low‐ and medium‐economies follow, for example, Panama, Puerto Rico, Mexico, Dominican Republic, and Malaysia. Regarding industrial authorship, *Sanofi* (France, USA, Singapore) and *Tekada* (USA, Switzerland, Singapore) are at the top. The majority of articles were published in the *American Journal of Tropical Medicine and Hygiene* (*n*_CT_ = 26), Vaccine (*n*_CT_ = 24), and *Journal of Infectious Diseases* (*n*_CT_ = 3) (Table 3). The complete list can be found in Supporting Information S1.

**TABLE 3:** Data on DENV_VAC_ publications from clinical trials (n_CT_). * = Country of origin of the authors without those from the industry, listed separately with the respective country, IF = Impact Factor.

Authors Country*	n_CT_ (authors' countries)	Authors Industry (country)	n_CT_ (Industry's countries)	Journal	n_CT_ (Journals)	If (2023)
USA	77	Sanofi (France)	40	Am J trop med Hyg	26	1.9
Thailand	36	Sanofi (USA)	33	Vaccine	24	4.5
Philippines	17	Tekada (USA)	23	J infect dis	23	5.0
Colombia	17	Tekada (Switzerland)	19	Hum vaccin immunother	11	4.1
Brazil	15	Sanofi (Singapore)	18	Pediatr infect dis J	10	2.9
Panama	11	Tekada (Singapore)	11	Lancet infect dis	9	36.4
Puerto Rico	11	Sanofi (Mexico)	9	PLoS Negl trop dis	6	3.4
Mexico	9	Sanofi (Uruguay)	9	Lancet	5	98.4
Singapore	7	Sanofi (Colombia)	8	Clin infect dis	4	8.2
Domenican rep.	7	GSK (Belgium)	8	Hum vaccin	4	—
Sri Lanka	6	Sanofi (China)	7	N Engl J med	4	96.2
Australia	6	Tekada (Brazil)	5	PLoS one	2	2.9
Honduras	6	GSK (USA)	5	BMC inf dis	2	3.4
Vietnam	6	Sanofi (Brazil)	4	EBioMedicine	1	9.7
Nicaragua	5	Merck (USA)	3	Front immunol	1	5.7

The majority of n_CT_ were funded by industry and US government organizations: *Sanofi*, France (*n* = 58), *Takeda*, Japan (*n* = 26), US Army (*n* = 17), and NIAID, USA (*n* = 17). Smaller amounts came from the Brazilian *Butantan Institute* (*n* = 4) and Merck, USA (*n* = 3). Other sponsors funded only one clinical trial that resulted in an article (Australian Defence Force, *Emergex Vaccines* (UK), *Inviragen* (USA), *Serum Institute* (India), *University of Maryland* (USA), and the WHO.

**FIGURE 7:**
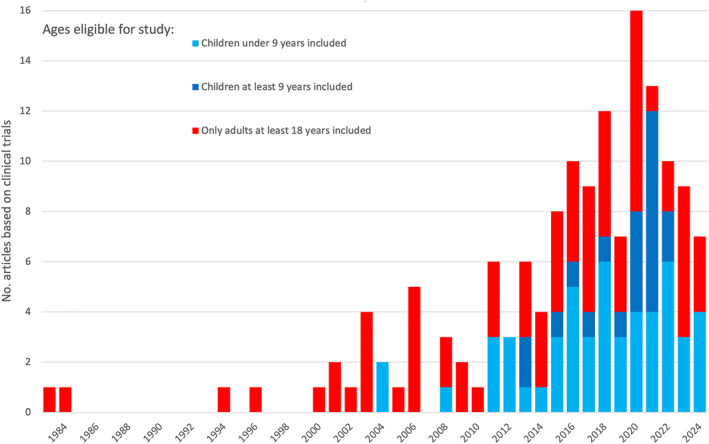
Development of DENV_VAC_ publications based on clinical trials, sorted by the lowest age eligible for the study.

The first publications on clinical DENV_VAC_ trials were published in 1983 and 1984 and covered only adults. The first article to include young children (< 9 years) was in 2004, but the inclusion of older children (≥ 9 years) began later and, together with younger children, reached the majority of publications in 2021 and 2022 (Figure 7).

## 4 Discussion

This study analyzes and evaluates the overall publication output on DENV_VAC_ from various aspects, ranging from chronological to geographical influences, from specific perspectives that include demographic, economic, and clinical patterns.

The development of DENV_VAC_ research and publication is, in principle, closely linked to vaccine development, its complexities and requirements, and the regional circumstances that make it necessary. Different quandaries impeded the vaccine development. The interaction of the four serotypes, the lack of a suitable animal model, and the missing knowledge about correlates as measurable characters for protection and disease enhancement, such as antibodies, hindered progress. [42].

As DENV mainly occurs in low‐ and middle‐income countries, the interest of high‐income countries in research funding was rather low. The reasons for this are the limited assessment of the global disease burden and the vaccine market, which was also considered limited. [43].

### 4.1 Chronological Aspects

As a result, overall research interest remained limited until the 2000s, despite efforts to develop dengue vaccines dating back to the 1940s [44] This can be shown by the annually published DENV_VAC_ articles of this study. The first article to be included in the analysis database dates back to 1945, when the *US Army Epidemiological Committee* analysed inoculated prisoners who had volunteered for vaccine development against dengue fever. [45] The number of articles remained correspondingly low until US researcher Thomas Chambers constructed the first vaccine in 1997, a chimaeric yellow fever DENV‐2 vaccine. [46] This discovery, combined with the accelerated global spread of the vectors *Ae. Aegypti* and *Ae. Albopictus* has changed the situation considerably. The resulting increasing awareness of dengue fever and access to new molecular techniques led to an acceleration of research, [47] which in turn led to Sanofi's first authorised vaccine (CYD‐TDV) in 2015. [48] This development is reflected in the steadily increasing number of articles since then. The emerging Zika virus, which raised international concern in 2016, was a further challenge for developing DENV_VAC_ due to the genetic similarity and potential immunological interaction between the two viruses. [49] Whether immunity to Zika protects against subsequent DENV infection or enhances it remains unclear. [44].

In 2019, CYD‐TDV was approved by the *US Food and Drug Administration* (FDA), but only for seropositive adolescents from endemic areas in the USA. It has also been authorised for a wider age group in Europe. [44] The WHO prequalified it in March 2020. [50] Sanofi is currently planning to discontinue production in 2026. It is currently only prescribed in Puerto Rico (USA), although no other vaccines have yet been approved in the USA. [51].

Subsequently, the COVID‐19 pandemic has certainly contributed to the diversion of research funds to studies on other zoonotic infections, resulting in a sharp decline in annual articles on DENV_VAC_. Similarly, previous studies showed a low level of interdisciplinary research in 2020. [52] Nevertheless, the global pandemic was also a stimulus for the development of dengue vaccines for their wider use, [44] reversing the declining trend in 2023. In May 2024, TAK‐003, which *Takeda* developed, received prequalification from the WHO. [50].

### 4.2 Geographical Aspects

The timely association of DENV_VAC_ research and vaccine development is also reflected in the geographical results of this study. The USA, France, Thailand, Singapore, and Brazil are at the top of the absolute ranking. First of all, the USA maintains its position as the leading country in research activity in this respect as well. From 49 continental US states with dengue infections, six states reporting locally acquired cases, and six endemic territories, [53] DENV awareness in the USA is high. However, the USA's interest in DENV research was also based on the massive troop movements to South Asia and the Pacific during the Second World War (WWII). DENV infections, together with malaria, led to a massive reduction in troop strength and, therefore, posed a formidable challenge to the US Army. [54] To date, the *Walter Reed Army Institute of Research* (WRAIR) is one of the most published institutions on DENV_VAC_ and, together with the NIH, is involved in developing the new candidates TV‐003 and TV‐005. [55].

After WWII, rapid urbanisation in Southeast Asia provided a favourable background for the increase in DENV infections due to the urban preferences of the DENV vectors, [56] leading to epidemics in India, the Philippines, Thailand, Malaysia, Singapore, Vietnam, Indonesia, and Myanmar. [54, 57] Some of these countries are the collaborating nations or subsidiary countries of the leading pharmaceutical companies in the context of DENV_VAC_ research. Initially, efficacy studies were mostly conducted in the USA, where adults were experimentally infected with qualified DENV strains before large‐scale field trials were initiated. [57] In particular, the early collaboration of *Sanofi* with *Mahidol University* in Thailand played an important role in developing and testing CYC‐TDV in large‐scale trials, especially in children. [58] This role can be confirmed by Mahidol University's leading position in the ranking of institutions engaged in DENV_VAC_ research. The necessity of the inclusion of endemic countries in those large‐scale trials is obvious. However, in India, there is currently no vaccine licenced. The Indian pharmacological company *Panacea Biotec* and the *Council of Medical Research* (ICMR) have launched the first Phase 3 clinical trial for an indigenous Indian tetravalent DENV vaccine candidate (*DengiAll*) developed with the collaboration of the US NIH. [59].

In the 1970s and 80s, dengue fever infections shifted from Asia to the Americas, where cases increased steadily. [57] The largest outbreaks occurred in Brazil, but other South American countries were also affected. [27] DENV fever first appeared in Europe in 1979 and in the continental USA in 1985. This regional development shows the involvement of the affected countries in research over time in a correspondingly increasing or decreasing manner. Brazil currently has the highest absolute number of cases per country (not the incidence rate, which is higher in Southeast Asia). This explains Brazil's commitment to DENV_VAC_ research, with which it will already be in third place behind the USA and India in the first half of 2024. In Brazil, where a large proportion of DENV_VAC_ research funding also comes from the government, the commitment to research is very high. The world's largest epidemic is expected to occur in 2024 in Brazil. Brazil has become the first country to integrate TAK‐003 into its public health system, and its DENV vaccine, *Butantan*, will soon be available. [60] Originally developed by the US NIH, Brazil has finalised *Butantan*. It is a one‐dose vaccine and is cheaper than TAK‐003. Although there is currently no evidence of antibody‐dependent enhancement, further follow‐up is needed to ensure safety.

India, which represents the Southeast Asian burden of dengue infections in the context of high incidence rates, has moved up to second place in publication numbers in the first half of 2024, demonstrating its high engagement in DENV_VAC_ research.

Spain and Uruguay achieved remarkable citation rates for their publication on DENV_VAC._ Although it is classified as a high‐income country, Uruguay is also the leader in R_GDP_ ranking. With the co‐authorships of both countries on highly cited efficacy studies according to their membership in the *Sanofi* and *Takeda* vaccine working groups, they were, therefore, able to achieve first places in the ranking of national citation rates, for example, 1. Sanofi Collaboration of 13 countries: CYD‐TDV Working group. 2. Takeda Collaboration of 11 countries, TIDES Working group (*Tetravalent Immunisation against Dengue Efficacy Study*). [32, 61].

### 4.3 Clinical Trials

In general, all clinical trials were conducted in Asia or Latin America: Thailand, India, Peru, and Mexico (Phase 2), and Brazil, Mexico, Colombia, Honduras, Puerto Rico, Philippines, Vietnam, Malaysia, Thailand, and Indonesia (Phase 3).

Studies based on clinical trials are among the most cited publications. Behind the USA, regarding the number of those publications, Thailand follows, which also ranks second in the R_GDP_ ranking. The Mahidol University and AFRIMS are highly effective institutions that achieved the highest citation rates.

The clinical studies resulting in publications on DENV_VAC_ were primarily conducted and authored by *Sanofi*. They were mostly published in average journals, but the high‐ranking published studies also achieved high citation numbers in *The Lancet* and the *New England Journal of Medicine* (NEJM). According to the trajectory of the studies, continuous publication started in the 2000s and peaked in 2020, which corresponds to the trajectory of all publications on DENV_VAC_.

In contrast, Japan, where *Takeda* is headquartered, is not among the countries with the most publications but performs well in terms of citation rate. This result is due to the highly cited clinical trial studies. Nevertheless, *Takeda* ranks only fifth in the institutions' analyses. The Japanese institutions also achieve a comparatively low citation rate.

### 4.4 Funding Aspects

National funding for DENV_VAC_ research is mainly provided by the USA, Brazil, France, China, and Japan, with more than 100 grants. The contribution of the pharmaceutical companies is clear, with Sanofi providing more than 85% of the French grants. In contrast, the Brazilian and Chinese governments awarded almost all national grants at an average rate of 1 and 1.4 grants per article, respectively. Although regionally high DENV fever numbers have been reported in China since 1978, gradually expanding to the largest outbreak in 2019, China does not consider DENV infections as important. [62] Hence, its involvement in DENV_VAC_ research is very small compared to other life science fields [30] and only began in 2006. China is not involved in the studies with an endemic study site function. The focus of participation is on the work of *Sanofi's* biometrics and biostatistics department in China and the university's collaboration with *Sanofi*.

Although some African countries are endemic to all four serotypes and active transmission of DENV has also been detected, the continent is almost unrepresented in DENV_VAC_ research. In the tropical parts of Africa, the climate and the extremely increasing and unplanned urbanisation provide optimal conditions for the vectors that led to the massive disease outbreak in 2023. However, most infections are not diagnosed at all or are misdiagnosed, meaning that the official figures are underestimated. Therefore, measures to contain the vectors in the context of urban development and the approval of vaccines are urgently needed here. [1] The difficulties in establishing surveillance must also be overcome to determine the actual burden.

## 5 Conclusions

The results of this study show that some regions where the dengue virus is endemic are severely underrepresented in dengue vaccination research. The increase in the proportion of high and very high human density areas and the high climate suitability for the vectors explain the recent increase in global dengue incidence. In the Global South, this increase was predominantly driven by population growth, while in the Global North, it was driven by an expansion in vector suitability to more temperate climates. [63] Dengue vaccination can and must complement, not replace, prevention methods, surveillance, and outbreak response tools to reduce the burden of the disease [48]. The clear regional concentration of DENV_VAC_ research is partly explained by the lack of access to reliable surveillance and serological data, but also by the regional interests of the pharmaceutical companies, their resources, and financial support. Therefore, international

Collaboration, for example, sharing of data, expertise, and resources, is crucial. [64].

## Author Contributions

**DK:** conceptualisation, data curation, investigation, methodology, formal analysis, supervision, visualisation, writing – original draft, writing – review and editing. **MB:** resources, validation, visualisation, writing – review and editing. **IMK:** resources, validation, visualisation, writing – review and editing. **CAN:** resources, visualisation, writing – review and editing. **DAG:** resources, software, validation, visualisation, writing – review and editing. **DB:** project administration, resources, writing – review and editing.

## Funding

Not applicable. No funding was received for this study.

## Conflicts of Interest

The authors declare no conflicts of interest.

## Supporting information


Supporting Information S1

## Data Availability

The datasets used and/or analyzed during the current study are available from the corresponding author upon reasonable request.
