# Supplementary material for: Characteristics, Motivation, and Challenges of Global Research on Dengue Vaccination
Source: Rev Med Virol. 2026 Mar 1;36(2):e70122. doi: 10.1002/rmv.70122 (PMC12950942; doi:10.1002/rmv.70122)
Supplement: Supplementary file 1 — Supporting Information S1 [file RMV-36-e70122-s001.docx]

Supplement

**Characteristics, Motivation, and Challenges of Global Research on Dengue Vaccination**

*Supplementary Table 1: Socio-economic indicators of countries with at least 10 publications on DENV_VAC_, GDP = gross domestic product, R_GDP_ = number of articles/GDP in 10 billion US dollars, R_POP_ = number of articles/population in millions of inhabitants, Countries economic categorization after World Bank* *(48): HI = high-income country, LMI = low-middle-income country, UMI = upper-middle-income country, Data 2022 from the UNESCO (49), except for Cuba (data from 2020, latest available data) and Taiwan (no data available), sorted by R_GDP_.*

| **Country** | **Articles** | **GDP** (10 bn)  US-$ | **Population** (mill.) | **R_GDP_** | **Rank  R_GDP_** | **R_POP_** | **Rank  R_POP_** |
| --- | --- | --- | --- | --- | --- | --- | --- |
| Uruguay | 27 | 7.12 | 3.42 | 3.79 | HI 1 | 7.89 | HI 3 |
| Thailand | 182 | 49.53 | 71.64 | 3.67 | UMI 1 | 2.54 | UMI 2 |
| Singapore | 155 | 46.68 | 5.96 | 3.32 | HI 2 | 26.02 | HI 1 |
| Sri Lanka | 20 | 7.44 | 21.80 | 2.69 | LMI 1 | 0.92 | LMI 1 |
| Panama | 14 | 7.65 | 4.38 | 1.83 | HI 3 | 3.20 | HI 5 |
| Philippines | 68 | 40.43 | 114.67 | 1.68 | LMI 2 | 0.59 | LMI 2 |
| Colombia | 57 | 34.39 | 51.79 | 1.66 | UMI 2 | 1.10 | UMI 4 |
| Malaysia | 65 | 40.63 | 33.75 | 1.60 | UMI 3 | 1.93 | UMI 3 |
| Dominican Rep. | 12 | 11.36 | 11.18 | 1.06 | UMI 4 | 1.07 | UMI 5 |
| Cuba | 39 | 0.00 | 11.22 | 1.05 | UMI 5 | 3.48 | UMI 1 |
| Vietnam | 43 | 40.88 | 97.84 | 1.05 | LMI 3 | 0.44 | LMI 3 |
| Switzerland | 75 | 80.77 | 8.71 | 0.93 | HI 4 | 8.61 | HI 2 |
| France | 235 | 278.29 | 64.56 | 0.84 | HI 5 | 3.64 | HI 4 |
| Brazil | 145 | 192.01 | 214.83 | 0.76 | UMI 6 | 0.67 | UMI 6 |
| Pakistan | 26 | 37.65 | 233.52 | 0.69 | LMI 4 | 0.11 | LMI 4 |
| Mexico | 74 | 141.42 | 127.02 | 0.52 | UMI 7 | 0.58 | UMI 7 |
| Belgium | 30 | 57.86 | 11.64 | 0.52 | HI 6 | 2.58 | HI 7 |
| South Korea | 82 | 166.52 | 51.83 | 0.49 | HI 7 | 1.58 | HI 10 |
| Portugal | 12 | 25.19 | 10.28 | 0.48 | HI 8 | 1.17 | HI 11 |
| UK | 120 | 307.07 | 67.39 | 0.39 | HI 9 | 1.78 | HI 9 |
| Indonesia | 48 | 131.91 | 274.62 | 0.36 | UMI 8 | 0.17 | UMI 9 |
| USA | 873 | 2546.27 | 337.50 | 0.34 | HI 10 | 2.59 | HI 6 |
| Australia | 54 | 167.54 | 26.05 | 0.32 | HI 11 | 2.07 | HI 8 |
| India | 106 | 338.51 | 1412.32 | 0.31 | LMI 5 | 0.08 | LMI 6 |
| Bangladesh | 13 | 46.02 | 170.30 | 0.28 | LMI 6 | 0.08 | LMI 5 |
| Argentina | 12 | 63.28 | 45.39 | 0.19 | UMI 9 | 0.26 | UMI 8 |
| Canada | 37 | 213.98 | 38.29 | 0.17 | HI 12 | 0.97 | HI 12 |
| Saudi Arabia | 17 | 110.81 | 36.14 | 0.15 | HI 13 | 0.47 | HI 15 |
| Germany | 57 | 407.22 | 83.43 | 0.14 | HI 14 | 0.68 | HI 13 |
| Japan | 51 | 423.11 | 124.28 | 0.12 | HI 15 | 0.41 | HI 16 |
| Netherlands | 11 | 99.11 | 17.54 | 0.11 | HI 16 | 0.63 | HI 14 |
| Spain | 10 | 139.75 | 47.58 | 0.07 | HI 17 | 0.21 | HI 17 |
| Italy | 12 | 201.04 | 59.12 | 0.06 | HI 18 | 0.20 | HI 18 |
| China | 79 | 1796.32 | 1425.93 | 0.04 | UMI 10 | 0.06 | UMI 10 |

*Supplementary Table 2: Data on publications of clinical trials on dengue vaccines with the number of authorships per country, company, and journal, IF = Impact Factor of 2023, * The number of authorships per country of origin is calculated without the private pharmaceutical companies involved in vaccine production, which are listed separately.*

| **Authors Country** | **Articles** | **Authors Company** | **Country** | **Articles** | **Journal** | **Articles** | **IF (2023)** |
| --- | --- | --- | --- | --- | --- | --- | --- |
| USA | 77 | Sanofi | France | 40 | Am J Trop Med Hyg | 26 | 1,9 |
| Thailand | 36 | Sanofi | USA | 33 | Vaccine | 24 | 4,5 |
| Philippines | 17 | Tekada | USA | 23 | J Infect Dis | 23 | 5,0 |
| Colombia | 17 | Tekada | Switzerland | 19 | Hum Vaccin Immunother | 11 | 4,1 |
| Brazil | 15 | Sanofi | Singapore | 18 | Pediatr Infect Dis J | 10 | 2,9 |
| Panama | 11 | Tekada | Singapore | 11 | Lancet Infect Dis | 9 | 36,4 |
| Puerto Rico | 11 | Sanofi | Mexico | 9 | PLoS Negl Trop Dis | 6 | 3,4 |
| Mexico | 9 | Sanofi | Uruguay | 9 | Lancet | 5 | 98,4 |
| Singapore | 7 | Sanofi | Colombia | 8 | Clin Infect Dis | 4 | 8,2 |
| Domenican Rep. | 7 | GSK | Belgium | 8 | Hum Vaccin | 4 | – |
| Sri Lanka | 6 | Sanofi | China | 7 | N Engl J Med | 4 | 96,2 |
| Australia | 6 | Tekada | Brazil | 5 | PLoS One | 2 | 2,9 |
| Honduras | 6 | GSK | USA | 5 | BMC Inf Dis | 2 | 3,4 |
| Vietnam | 6 | Sanofi | Brazil | 4 | EBioMedicine | 1 | 9,7 |
| Nicaragua | 5 | Merck | USA | 3 | Front Immunol | 1 | 5,7 |
| Malaysia | 5 | Sanofi | Thailand | 3 | Hum Immunol | 1 | 3,1 |
| France | 4 | Sanofi | Philippines | 3 | J Clin Invest | 1 | 13,3 |
| UK | 3 | Sanofi | Belgium | 1 | J Virol | 1 | 4,0 |
| Uruguay | 3 | Vical Inc. | USA | 1 | Lancet Glob Health | 1 | 19,9 |
| Indonesia | 3 |  |  |  | mSphere | 1 | 3,7 |
| Switzerland | 2 |  |  |  | Nature Comm | 1 | 14,7 |
| India | 2 |  |  |  | PLoS Pathog | 1 | 5,5 |
| China | 2 |  |  |  | Proc Natl Acad Sci USA | 1 | 9,4 |
| Peru | 2 |  |  |  | Sci Transl Med | 1 | 15,8 |
| Germany | 1 |  |  |  | Trans R Soc Trop Med Hyg | 1 | 1,9 |
| Bangladesh | 1 |  |  |  |  |  |  |
| Cambodia | 1 |  |  |  |  |  |  |
| Belgium | 1 |  |  |  |  |  |  |
| Saudi Arabia | 1 |  |  |  |  |  |  |
| Canada | 1 |  |  |  |  |  |  |
| Paraguay | 1 |  |  |  |  |  |  |
| South Korea | 1 |  |  |  |  |  |  |
